# Pediatric Innominate Artery Pseudoaneurysm Rupture in Vascular Ehlers-Danlos Syndrome: A Case Report

**DOI:** 10.5811/cpcem.2021.3.51787

**Published:** 2021-04-23

**Authors:** Aimee Vos, Katharine M. Burns

**Affiliations:** Advocate Christ Medical Center, Department of Emergency Medicine, Oak Lawn, Illinois

**Keywords:** Pseudoaneurysm, pediatric vascular injury, Ehlers-Danlos syndrome, case report

## Abstract

**Introduction:**

Ehlers-Danlos syndrome is a well classified connective tissue disorder recognized by its features of hyperextensibility of joints and hyperelasticity of the skin. However, the rare vascular type (Ehlers-Danlos type IV) is more difficult to identify in the absence, rarity, or subtlety of the classical physical features. Patients presenting to the emergency department (ED) with acute complications of vascular Ehlers-Danlos syndrome may be critically ill, requiring accurate diagnosis and tailored management.

**Case Report:**

This report details a case of spontaneous innominate artery pseudoaneurysm rupture in a pediatric patient with previously undiagnosed Ehlers-Danlos syndrome. Initial ED evaluation was followed by urgent operative intervention and subsequent genetic testing to confirm final diagnosis.

**Conclusion:**

Due to its high morbidity and mortality, vascular type Ehlers-Danlos syndrome should be considered in the differential for otherwise unexplained spontaneous vascular injury.

## INTRODUCTION

Ehlers-Danlos syndrome (EDS) is a broad class of connective tissue disorders unified by a variable degree of joint hypermobility and skin involvement, including fragility, hyperelasticity and/or bruising.^1^ The various forms of EDS can be distinguished on the basis of clinical presentation, inheritance (generally autosomal dominant), the altered gene, and the underlying mechanism that renders extracellular collagen deficient. The vascular type of EDS (vEDS) is a rare condition with an estimated prevalence of one in 50,000–250,000 individuals.^2^,^3^ Characteristic features of vEDS include fragility of the arteries, intestine, and uterus. Outward features often include a characteristic facial appearance (deeply set and prominent eyes, narrow nasal bridge, thin lips), translucent skin with dystrophic scars, frequent occurrence of bruising without an identified cause, and tapered fingers with an aged appearance known as acrogeria.^2^

Notably absent in vEDS is the prominent joint hypermobility typically associated with the more common hypermobile, or classical, presentations of EDS.^3^,^4^,^5^ If present in vEDS, joint laxity is often limited to the distal extremities. Less consistent features include clubfoot deformity, spontaneous pneumothorax, and cavernous sinus fistula. Unlike all other forms of EDS, vEDS is caused by mutations in the gene (*COL3A1*) encoding type III collagen.^1^

The diagnosis of vEDS in childhood most often occurs as a consequence of a dedicated clinical evaluation and molecular testing after diagnosis of a family member. The majority of additional diagnoses occur on the basis of a life-threatening vascular or gastrointestinal event, highlighting the need for increased clinical awareness of the signs and symptoms of this disorder. This report details a pediatric patient with a spontaneous innominate artery pseudoaneurysm rupture serving as the inciting incident, leading to his formal diagnosis of vEDS.

## CASE REPORT

A 15-year-old male presented to the emergency department for evaluation of neck and chest pain. He was running at a brisk pace when he felt a sudden sharp pain in his neck followed by sustained pain in the center of his chest and neck. He reported the sensation of shortness of breath and nausea, but no dysphagia or dysphonia. There was no history of trauma to the head, neck, or chest. He was previously healthy with no prior medical problems or prior surgeries and had no history of familial or congenital medical conditions. Most notably, the patient had no family history of sudden cardiac death, aortic disease, or connective tissue disease. The patient endorsed having no known allergies and did not take any medications on a daily basis.

On arrival, he displayed mild tachypnea with a respiratory rate of 22 breaths per minute, but otherwise had normal vital signs with a blood pressure of 128/80 millimeters of mercury, heart rate 66 beats per minute, temperature 37 degrees Celsius, with 98% oxygen saturation on room air. His airway showed no evidence of impending compromise. He appeared to be in moderate distress secondary to pain. He demonstrated respiratory splinting and held his neck and shoulders still to prevent worsening of pain. He had mild nasal flaring, but his lungs were clear to auscultation bilaterally. He had no evidence of stridor. Palpation of the chest revealed tenderness near the right sternoclavicular region of his chest. Pulses were palpable and equal in all extremities.

**Image 1:**
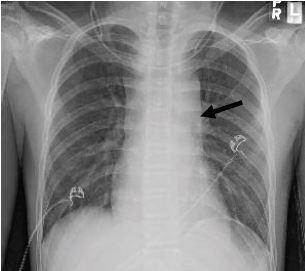
Upright chest radiograph showing a widened mediastinum (arrow) in a 12-year-old with vascular Ehlers-Danlos Syndrome.

**Image 2:**
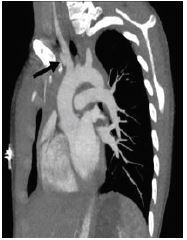
Pseudoaneurysm (arrow) of the innominate artery as it extends off of the arch of the aorta, as seen in the coronal axis on computed tomography angiogram imaging of a 12-year-old with vascular Ehlers-Danlos Syndrome.

**Image 3:**
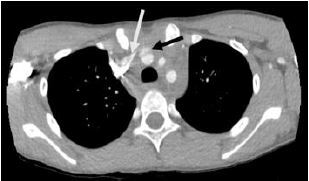
Pseudoaneurysm of the innominate artery (black arrow) seen in the transverse axis on computed tomography angiogram imaging with contrast. Mediastinal hematoma (white arrow) is seen layering anterior to the hyperdense subclavian vein as contrast extravasates out of the ruptured pseudoaneurysm.

A chest radiograph showed lung markings to the periphery bilaterally, no consolidations, and no opacities (Image 1). The mediastinum appeared widened, which prompted a computed tomography (CT) angiogram of the chest and neck to better evaluate vascular anatomy. This revealed a pseudoaneurysm of the proximal innominate artery (Image 2). There was also high attenuation blood tracking from the superior mediastinum to the descending aorta suggesting rupture of the pseudoaneurysm and formation of a mediastinal hematoma (Image 3).

**CPC-EM Capsule**

What do we already know about this clinical entity?

*Vascular type Ehlers-Danlos syndrome (EDS), a genetic connective tissue disorder, is characterized by vascular and organ fragility.*

What makes this presentation of disease reportable?

*Vascular type EDS can present initially with critical and potentially devastating organ or vascular injuries.*

What is the major learning point?

*Vascular type EDS should be considered in the differential for otherwise unexplained spontaneous vascular injury.*

How might this improve emergency medicine practice?

*Prompt diagnosis of vascular type EDS will significantly influence medical decision-making, allowing tailored care to the underlying pathology.*

The patient was taken to the operating room where he was found to have an extensive tear of the proximal innominate artery about one centimeter from its origin. The rupture was contained by the adventitia and surrounding soft tissue. The patient received an ascending aortic to innominate artery extra-anatomic bypass graft, and drains were placed to reduce the mediastinal hematoma. He was evaluated by geneticists postoperatively who suspected a connective tissue disorder. High on the differential was vEDS given his exam exhibiting flexible digits, easy bruising, and normal aortic root dimension. The patient was discharged home on postoperative day five, and nearly three weeks later, genetic testing confirmed a pathogenic *COL3A1* mutation diagnostic of vEDS.

## DISCUSSION

The Ehlers-Danlos phenotype was first described by Van Meekeren in 1682 after reporting a patient with hyperelastic skin. It was not until Ehlers and Danlos further classified the additional classical characteristics in the early 1900s that the collection of signs and symptoms became recognized as a syndrome.^6^ Throughout the 1960s the syndrome was further classified into distinct types.^4^ Finally in 1997, after up to 11 types had been described, the Villefranche nosology classified the syndrome into the six types commonly referenced in medicine today: classical; hypermobility; vascular; kyphoscoliosis; arthrochalasia; and dermatosparaxis.^7^,^8^ These classifications were based on clinical phenotype, inheritance pattern, and biochemical and molecular defects.^3^ Vascular type, otherwise known as type IV, is rare but also the most malignant of the six types.

Clinical diagnosis of vEDS includes two of the four main criteria: 1) easy bruising; 2) thin skin and visible veins; 3) facial characteristics including prominent eyes, narrow nasal bridge, and thin lips; and 4) uterine, arterial, or intestinal rupture.^3^,^5^ Factors favoring this diagnosis in the described case include the young age associated with vascular event and the absence of aortic root enlargement, which is a common finding in other vascular connective tissue diagnoses including Marfan syndrome and Loeys-Dietz syndrome. Laboratory diagnosis is used to confirm clinical suspicion. Identification of a pathogenic *COL3A1* mutation shows great sensitivity and specificity for the diagnosis of vEDS. Approximately half of cases are due to new mutations that occur sporadically with no family history of connective tissue disorder.^3^

The morbidity of vEDS is quite high. In a review of 220 index patients and 199 affected relatives performed by Pepin et al in 2000, median survival was reported to be 48 years, but a quarter of patients had their first complications before 20 years of age, with average age of first complication 23.5 years. At the age of 40, 80% of patients reported having at least one major complication including arterial dissection or rupture (46%), gastrointestinal perforation (19%), or other organ rupture (5%). Arterial events are associated with a high rate of fatality.^5^ Common sites of initial arterial events include the aortic arch and branch vessels or the descending aorta in the chest or abdomen. Less common sites for arterial rupture include the carotid, subclavian, ulnar, popliteal, and tibial arteries.^7^ This report describes an innominate artery pseudoaneurysm defined as an incomplete vascular rupture extending through the intima and media, but contained by the adventitia and surrounding soft tissue.

While there is no definitive treatment for vEDS, there are approaches that can be used to reduce morbidity and mortality including the use of high-dose vitamin C to promote collagen cross-linking, antihypertensive agents to reduce hemodynamic stress and to mitigate abnormal cellular signaling events in the arterial wall, and frequent noninvasive imaging to identify emerging vascular lesions.^3^ Pregnancy is considered high risk given the potential for uterine rupture and should be managed by high-risk specialists. Patients should be encouraged to avoid contact sports or other activities with risk of traumatic injury.^2^,^3^

Invasive procedures such as angiography should be avoided in patients with vEDS if possible, as pseudoaneurysm formation, dissection, or vessel rupture has been associated with arterial manipulation.^3^,^6^,^9^ If angiography is necessary for diagnosis in a symptomatic patient, contrast injection should be performed at low pressure to avoid injury to the vessels. Caution is indicated with regard to endovascular diagnostic procedures or therapy given the potential for vascular fragility or rupture. As a general precaution, stent procedures are avoided in vEDS due to the potential for vessel rupture or stent migration.^3^ The benefit of prophylactic surgical intervention in vEDS must be weighed against the risks imposed by tissue fragility. Conservative management is typically favored unless a potentially fatal complication is identified.^2^

## CONCLUSION

In the ED, young patients with vEDS can present with potentially catastrophic complications prior to receiving a formal diagnosis of a connective tissue disorder. Emergency physicians need to be wary of complaints of chest pain or abdominal pain in patients with known or suspected vEDS as this may be the presenting complaint due to a severe vascular injury. Even if symptoms appear minor, vascular imaging is crucial to detecting underlying injury. Angiography and operative intervention, while risky, may be necessary if a life-threatening vascular abnormality or hemorrhage is identified. If vascular injury is ruled out, the emergency physician should have a high index of suspicion for other associated complications including bowel rupture, uterine rupture, or spontaneous pneumothorax. The morbidity and mortality of these complications could be mitigated by an increased awareness of systemic manifestations and an appropriate clinical suspicion by both primary care and emergency physicians.
